# Clonal Integration Promotes the Photosynthesis of Clonal Plant Under Heterogeneous Pb and/or Pyrene Stress

**DOI:** 10.3390/toxics12120899

**Published:** 2024-12-11

**Authors:** Yichen Liu, Sunan Xu, Xuemei Li, Lihong Zhang

**Affiliations:** 1 School of Environment, Liaoning University, Shenyang 110036, China; lychennn@126.com (Y.L.); xusunan@lnu.edu.cn (S.X.); 2 College of Life Science, Shenyang Normal University, Shenyang 110034, China; lxmls132@163.com

**Keywords:** chlorophyll fluorescence, heavy metals, heterogeneous treatment, PAHs, photosynthetic parameters

## Abstract

Clonal plants can support the growth of their ramets in heterogeneous environments through clonal integration between the ramets. However, the role of clonal integration in modulating ramet photosynthesis under toxic stress, especially combined stress, is unclear. This study examines the impact of clonal integration on *Zoysia japonica* under three heterogeneous stresses (Pb, pyrene, and Pb+Pyrene) with two stolon connection conditions (connected and disconnected). Our results show that clonal integration significantly enhances P_N_, g_s_, C_i_, E, and CE while reducing WUE. It also improves ΦPSII, F_v′_/F_m′_, F_v_/F_m_, F_v_/F_0_, and q_P_ while reducing NPQ. Clonal integration lowers MDA levels, increases SOD activity, and mitigates the decline in CAT and POD activity, resulting in increased biomass under stress. Furthermore, we observed that the synergistic effects of the Pb+Pyrene mixture negatively impacted the adaptability of clonal integration. Our study underscores the role of clonal integration in maintaining photosynthesis and supporting the success of clonal plants in toxic environments.

## 1. Introduction

Heavy metal pollution, particularly in soil, is a significant ecological issue that poses risks to human health [1]. Pb is the most common heavy metal and ranks as the most hazardous among 20 priority pollutants [2]. As a nonessential element, excessive Pb inhibits plant growth, reduces photosynthetic efficiency, disrupts cell membranes, and impairs PSII function [3]. Additionally, it is often found in coexistence with polycyclic aromatic hydrocarbons (PAHs) in soil, with about 90% of PAHs found in this environmental medium [4,5]. Pyrene, a type of PAH, is a priority pollutant due to its persistence and high toxicity. It can be absorbed by plants, affecting their antioxidant and photosynthetic systems and limiting biomass accumulation [6,7]. Importantly, the co-occurrence of heavy metals and PAHs can lead to interactive cytotoxic effects, posing significant challenges for plants in contaminated soil [4,8].

Photosynthesis is crucial for plant growth but is sensitive to contamination. This sensitivity makes it a common focus for studying how plants adapt to adverse conditions [9]. Recently, the use of chlorophyll fluorescence assays has grown due to their noninvasive nature, sensitivity, speed, and ease of operation [10]. Under Pb stress, mulberry seedlings show a reduced net photosynthetic rate (P_N_), stomatal conductance (g_s_), transpiration rate (E), and intercellular CO_2_ concentration (C_i_), along with a decreased maximal quantum yield of PSII (F_v_/F_m_), actual photochemical quantum efficiency of PSII (ΦPSII), and electron transport rate (ETR), while non-photochemical quenching (NPQ) increases [11]. Similarly, tomato plants exposed to pyrene stress exhibit significant declines in P_N_, g_s_, E, C_i_, F_v_/F_m_, ΦPSII, and the photochemical quenching coefficient (q_P_) [12].

The impact of soil co-stressed with heavy metals and PAHs has been extensively researched. However, the findings from these studies are inconsistent. For example, under co-stress conditions of Cd and pyrene, *Festuca arundinacea* shows higher biomass accumulation compared to when exposed to the individual stress of Cd [8]. Conversely, pyrene significantly increased Cu toxicity in *Brassica juncea* [13]. Moreover, pyrene mitigated growth inhibition caused by Cu in maize [14] and Pb in ryegrass [15]. However, it could not alleviate Cd toxicity in maize [16]. Thus, the response of plants to co-stress requires further analysis to understand the potential synergistic or antagonistic effects of these pollutants.

Clonal plants, commonly found in natural habitats, benefit from clonal integration, which allows for resource sharing among interconnected ramets (or offshoots) through their stolons (or rhizomes) [17]. This integration is a crucial adaptive strategy that helps clonal plants respond to environmental stress, significantly enhancing the fitness of individual ramets [18]. *Zoysia japonica* (Zoysia), a resilient clonal plant with an extensive root system, can survive in challenging environments [19]. Our research group has previously studied the clonal integration of Zoysia under heterogeneous nutrient conditions, discovering that this integration can significantly enhance P_N_, E, and g_s_ in connected stressed ramets [20]. Additionally, it can increase the activity of superoxide dismutase (SOD), peroxidase (POD), and catalase (CAT), while reducing malondialdehyde (MDA) levels [21]. Similar studies indicate that clonal integration can improve photosynthesis in stressed ramets under various stress conditions, such as changes in water and nitrogen availability [22], salinity stress [23], and submergence [24].

Resource distribution in natural environments varies, and pollutants also exhibit heterogeneous distributions [25]. Pollution levels can vary significantly after soil heavy metal contamination; some ramets may thrive in unpolluted microhabitats while remaining connected to those in contaminated areas [26]. Similarly, the distribution patterns of PAHs are heterogeneous in contaminated environments [27]. Moreover, considering the significant overlap between heavy metal and PAH sources and sinks [28], it is reasonable to suggest that clonal plants frequently encounter situations analogous to those in heterogeneous heavy metal soils. Specifically, some ramets may thrive in unpolluted microhabitats, while others face PAH pollution or are in areas contaminated by both heavy metals and PAHs. Previous studies have mainly focused on the advantages of clonal integration in the context of heterogeneous heavy metal pollution [17,29,30,31]. However, the effects of clonal integration in clonal plants subjected to heterogeneous PAH contamination, particularly when occurring simultaneously with heavy metal pollutants, remain largely unexplored.

In recent years, rapid industrial and agricultural modernization has led to severe soil contamination by heavy metals and PAHs [1,4]. Although considerable research has examined the effects of co-contamination on plant responses, the specific impacts on clonal plants under simultaneous exposure to these stressors remain underexplored. Most studies have focused on clonal integration related to heavy metal stress, highlighting a gap in understanding how clonal plants respond to both Pb and pyrene. This study aims to fill this gap by investigating the clonal integration of photosynthesis in Zoysia under heterogeneous Pb+Pyrene stress. We measured the biomass, chlorophyll content, photosynthetic parameters, chlorophyll fluorescence, MDA levels, and antioxidant enzyme (SOD, CAT, POD) activities in connected and disconnected ramets. By comparing the performance of clonal integration under heterogeneous combined stress and individual stress, we aim to gain deeper insights into the mechanisms of clonal integration.

## 2. Materials and Methods

### 2.1. Plant Materials and Experimental Treatments

The experiment was conducted at the Eco-Experimental Garden of Liaoning University, where soil was collected and mixed with sand in a ratio of 1:1 (Wsoil: Wsand). The basic properties of the mixed soil were as follows: pH, 7.23; total organic carbon, 8.8%; available nitrogen, 110 mg·Kg^−1^; available phosphorus, 62.5 mg·Kg^−1^; available potassium, 220 mg·Kg^−1^; Mg, 22 mg·Kg^−1^; Cr, 0.13 mg·Kg^−1^; Ni, 0.02 mg·Kg^−1^; Cu, 2.8 mg·Kg^−1^; Pb, 8.2 mg·Kg^−1^; Zn, 15.3 mg·Kg^−1^; Mn, 3.3 mg·Kg^−1^; and Cd, 0.05 mg·Kg^−1^.

Clonal ramets of similar size were selected, each containing two rooted ramets connected by a stolon. A small opening was made at the top of each plastic pot for the stolons. Soil moisture was maintained above 75% of field capacity through regular watering with distilled water. Additionally, a compound fertilizer containing 6.0% total organic nitrogen, 35% organic carbon, and 4% P_2_O_5_ (provided by Shenyang Zhongze New Fertilizer Co., Ltd., Shenyang, China) was applied every 14 days.

**Figure 1:**
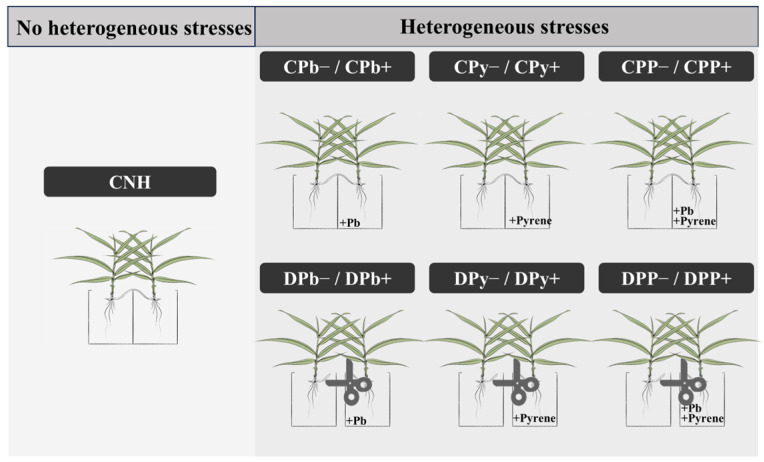
Experimental design. Connected and disconnected ramets of Zoysia were subjected to heterogeneous Pb and/or pyrene stress.

After a two-week recovery period, plastic pots were transferred to the garden. The garden’s environmental conditions were maintained at 18.5 ± 2.0 °C to 30.5 ± 2.0 °C, with a relative humidity of 60 ± 5%. After a normal growth period of 30 days in the garden, half of the clonal ramets were randomly chosen for stolon severance. After a 7-day recovery period, connected and disconnected ramets were treated with three heterogeneous stresses (Pb, pyrene, Pb+Pyrene). We adopted the soil contamination method from Lu et al. [8], which demonstrated excellent soil Pb recovery (101.2 ± 3.52%) and pyrene recovery (96.3 ± 6.32%). Using this method, we conducted soil contamination with Pb at 2.0 g·kg^−1^ and pyrene at 0.2 g·kg^−1^. The Pb (99%) and pyrene (98%) used in the experiment were sourced from Macklin Biochemical Co., Ltd. (Shanghai, China). This resulted in a total of 13 experimental treatments, as illustrated in Figure 1. For connected ramets, treatments included the following: no heterogeneous stress (CNH); no Pb stress (CPb−) and Pb stress (CPb+); as well as no pyrene stress (CPy−) and pyrene stress (CPy+); alongside no Pb+Pyrene stress (CPP−) and Pb+Pyrene stress (CPP+). Similarly, disconnected ramets underwent treatments of no Pb (DPb−) and Pb stress (DPb+), no pyrene (DPy−) and pyrene stress (DPy+), and no Pb+Pyrene (DPP−) and Pb+Pyrene (DPP+). Each treatment was replicated three times. After 4 days of treatment, ramets were harvested separately and weighed.

### 2.2. Determination of Chlorophyll Content

Chlorophyll content was measured using a chlorophyll meter (SPAD-502, Konica Minolta, Inc., Tokyo, Japan). The SPAD value was recorded as a relative indication of the total chlorophyll content in the leaves.

### 2.3. Determination of Photosynthetic Parameters

P_N_, g_s_, C_i_, and E were measured with a portable photosynthesis meter (Li-6400, Li-Cor, Lincoln, NE, USA) at a light intensity of 1000 μmol·m^−2^·s^−1^ and a CO_2_ concentration of 400 μmol·mol^−1^ at 09:00 A.M. to 11:00 A.M. Water-use efficiency (WUE) = P_N_/E, and carboxylation efficiency (CE) = P_N_/C_i_ [32].

### 2.4. Determination of Chlorophyll Fluorescence Parameters

Chlorophyll fluorescence parameters were measured using the Li-6400. The leaves were dark adapted for 30 min, and then the initial fluorescence (F_0_) and maximum fluorescence (F_m_) were measured. After that, the leaves were exposed to natural light for 60 min to activate them, and the steady-state fluorescence (F_s_), maximum fluorescence (F_m′_), and minimum fluorescence (F_0′_) were measured. The other fluorescence parameters were then calculated using the following formulas: F_v_/F_m_ = (F_m_ − F_0_)/F_m_; PSII potential activity, F_v_/F_0_ = (F_m_ − F_0_)/F_0_; PSII effective quantum efficiency, F_v′_/F_m′_ = (F_m′_ − F_0′_)/F_m′_; ФPSII = (F_m′_ − F_s_)/F_m′_; ETR = 0.5 × 0.85 × ФPSII × PPFD; q_P_ = (F_m′_ − F_s_)/(F_m′_ − F_0′_); and NPQ = (F_m_ − F_m′_)/F_m′_ [11,33].

### 2.5. Determination of Lipid Peroxidation and Antioxidant Enzyme Activity

Lipid peroxidation was estimated by measuring the content of MDA [34]. The content of MDA and the activities of SOD, POD, and CAT were determined using ELISA kits purchased from Jiangsu Meimian Industrial Co., Ltd. (Nanjing, China). The procedure and determination method were performed according to the instructions provided with the ELISA kit.

Leaf samples (1.0 g) were ground with liquid nitrogen, and then 9 mL of pre-cooled phosphoric acid buffer solution (pH = 7.3) was added. The mixture was centrifuged at 2500 rpm for 20 min at 4 °C. Finally, the supernatant was stored at −20 °C as the crude enzyme extract.

The ELISA kit operates on a double antibody sandwich method. Purified plant MDA/SOD/POD/CAT was placed in the microporous plate to form solid-phase antibodies. The crude enzyme extract was added to the microporous plate as the antigen. Then, horseradish peroxidase-conjugated second antibody was added to the microporous plate. Antigens and enzyme-labeled specific antibodies were combined on the solid-phase complex, and the unbound ones were removed after thorough washing. The substrate TMB was added to develop color, and then 50 μL of stop solution was added as a stop solution and finally appeared yellow. The optical density (OD) value of the samples at 450 nm was detected using a microplate reader (Infinite F50, Tecan Co., Ltd., Männedorf, Switzerland). The standard curve was established according to the OD values and concentrations of the standard samples, allowing for the calculation of MDA content and the activities of SOD, POD, and CAT through a linear regression equation.

### 2.6. Statistical Analysis

In this study, each experiment was independently repeated three times. Significant differences were determined using one-way analysis of variance (ANOVA) with the SPSS statistics (19.0) software. A significance level of *p* ≤ 0.05 was used to indicate significant differences, which were determined through the LSD test and two-tailed Student’s *t*-test. Figures were created using OriginPro 2021 and PROcreate programs.

## 3. Results

### 3.1. Growth Parameters and SPAD Value

**Figure 2:**
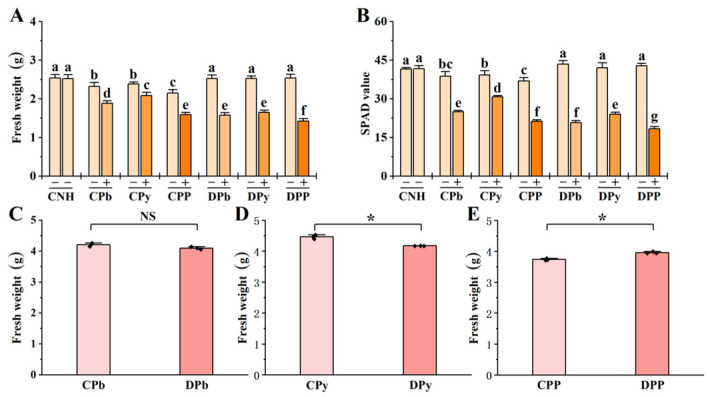
Growth parameters in connected and disconnected Zoysia ramets under heterogeneous stresses. Fresh weight (**A**), SPAD value (**B**), fresh weight of the whole clone under Pb treatment (**C**), fresh weight of the whole clone under pyrene treatment (**D**), and fresh weight of the whole clone under combined Pb+Pyrene treatment (**E**) graphs are shown. Fresh weights represent the total biomass per pot. Values are mean ± SD for *n* = 3. Bars with different letters are statistically different at *p* < 0.05, by LSD test (**A**,**B**). NS means not significant and * *p* < 0.05, by two-tailed Student’s *t*-test (**C**–**E**).

Stressed ramets showed significant reductions in fresh weight and SPAD values (Figure 2A,B). Clonal integration mitigated the inhibition in connected stressed ramets (CPb+, CPy+, and CPP+). Additionally, we observed that the benefits for CPP+ were less pronounced than those for CPb+ and CPy+. Disconnected unstressed ramets (DPb−, DPy−, and DPP−) were unaffected by heterogeneous stresses. In contrast, connected unstressed ramets (CPb−, CPy−, and CPP−) exhibited significant reductions in fresh weight and SPAD values, especially in CPP− where inhibition was most pronounced.

Moreover, our findings indicated that clonal integration conferred greater benefits than costs to the whole clone under individual Pb or pyrene stress (Figure 2C,D). Specifically, the fresh weight of CPb was greater than that of DPb, although the difference was not significant; however, CPy was significantly higher than DPy. Conversely, under the combined Pb+Pyrene stress, the costs associated with clonal integration for the whole clone outweighed the benefits, with the fresh weight of CPP being significantly lower than that of DPP (Figure 2E).

### 3.2. Photosynthetic Parameters 

**Figure 3:**
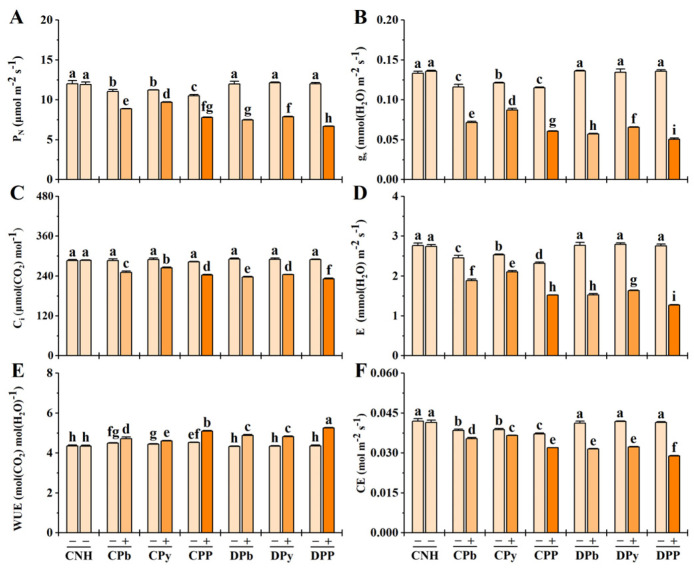
Photosynthetic parameters in connected and disconnected Zoysia ramets under heterogeneous stresses. P_N_ (**A**), g_s_ (**B**), C_i_ (**C**), E (**D**), WUE (**E**), and CE (**F**) graphs are shown. Values are mean ± SD for *n* = 3. Bars with different letters are statistically different at *p* < 0.05, by LSD test.

As depicted in Figure 3, the P_N_, g_s_, C_i_, E, and CE of stressed ramets significantly decreased under heterogeneous stresses, while the WUE increased. Connected stressed ramets (CPb+, CPy+, and CPP+) exhibited higher P_N_, g_s_, C_i_, E, and CE but lower WUE compared to disconnected treatments (DPb+, DPy+, and DPP+). Clonal integration had a limited effect on CPP+, in contrast to the more pronounced benefits observed in CPb+ and CPy+. Connected unstressed ramets (CPb−, CPy−, and CPP−) showed significant changes in P_N_, g_s_, E, WUE, and CE, with CPP− experiencing the highest impact.

### 3.3. Chlorophyll Fluorescence Parameters

**Figure 4:**
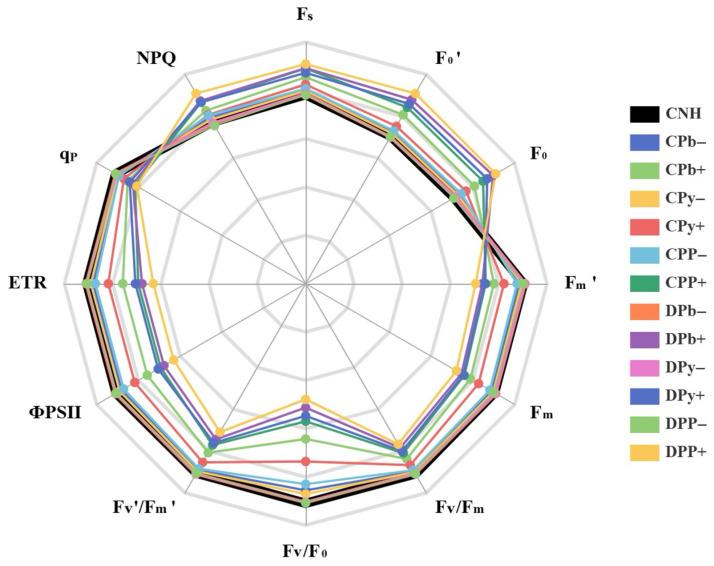
Spider chart of chlorophyll fluorescence parameters in connected and disconnected Zoysia ramets under heterogeneous stresses. Each line represents the average of 3 measurements per treatment. Each parameter is expressed as a fraction relative to the values of CNH (black line with a value 100% = 1).

Chlorophyll fluorescence tests provided a wealth of information. The stressed ramets exhibited significant declines in F_m′_, F_m_, F_v_/F_m_, F_v_/F_0_, F_v′_/F_m′_, ФPSII, ETR, and q_P_, while F_s_, F_0′_, F_0_, and NPQ increased (Figure 4). Among these, the most pronounced decrease compared to CNH was observed in F_v_/F_0_, followed by ФPSII and ETR. Notably, clonal integration mitigated these changes in connected stressed ramets (CPb+, CPy+, and CPP+), particularly enhancing F_v_/F_0_, ФPSII, and ETR. Conversely, the parameter least affected by clonal integration was q_P_. Importantly, its benefits were limited in CPP+ compared to those observed in CPb+ or CPy+. The parameters of connected unstressed ramets (CPb−, CPy−, and CPP−) were also affected, with CPP− experiencing the most significant impact.

### 3.4. Lipid Peroxidation and Antioxidant Enzyme Activity

**Figure 5:**
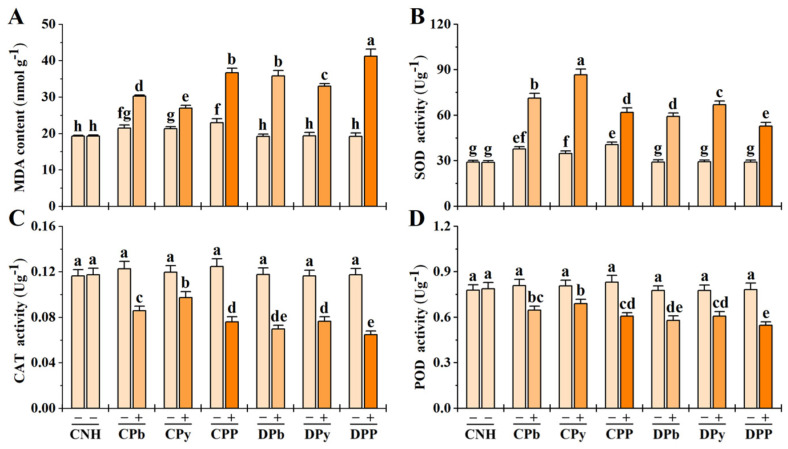
Lipid peroxidation and antioxidant enzyme activity in connected and disconnected Zoysia ramets under heterogeneous stresses. MDA content (**A**), SOD activity (**B**), CAT activity (**C**), and POD activity (**D**) are shown. Values are mean ± SD for *n* = 3. Bars with different letters are statistically different at *p* < 0.05, by LSD test.

MDA indicates membrane lipid peroxidation under heterogeneous stresses. In stressed ramets, MDA levels increased; however, connected treatments (CPb+, CPy+, CPP+) exhibited significantly lower MDA levels than disconnected treatments (DPb+, DPy+, DPP+) (Figure 5A). The positive effects of clonal integration were less pronounced in CPP+ compared to CPb+ or CPy+. Additionally, MDA levels notably increased in connected unstressed ramets (CPb−, CPy−, CPP−), particularly in CPP−.

In stressed ramets, SOD activity increased, while CAT and POD activities decreased (Figure 5B–D). Connected treatments (CPb+, CPy+, CPP+) further enhanced SOD activity and mitigated the decline in CAT and POD activities compared to disconnected ramets (DPb+, DPy+, DPP+). However, under CPP+, the increase in antioxidant enzyme activities due to clonal integration was lower than that observed under CPb+ or CPy+. Additionally, in connected unstressed ramets (CPb−, CPy−, CPP−), SOD levels increased significantly, while CAT and POD levels also improved, though not significantly.

## 4. Discussion

This study explores the impact of heterogeneous Pb and/or pyrene stress, revealing that the degree of growth inhibition varied among the stressed ramets. Importantly, clonal integration significantly increased the biomass accumulation of connected stressed ramets compared to disconnected ones. Additionally, pollution stress significantly reduces photosynthetic efficiency; however, this negative impact is mitigated through this integration. The ability to maintain photosynthesis can effectively support the resource demands of clonal plants in polluted environments. However, under conditions of combined heterogeneous pollution, the adaptive benefits of clonal integration are diminished.

Chlorophyll is crucial for photosynthesis but can be negatively impacted by abiotic stress. Maintaining high chlorophyll levels is essential for photosynthesis under stress [12]. This study found that clonal integration significantly increased SPAD values of stressed ramets. These results align with previous research, which showed that clonal integration under heterogeneous environments increases chlorophyll levels [35,36,37].

The most apparent sign of pollutant stress is a decrease in P_N_, which is directly related to carbon fixation efficiency and biomass accumulation [38]. Our data clearly showed that heterogeneous stresses cause a significant reduction in P_N_, while clonal integration alleviates this decline. Photosynthetic impairment often results from either stomatal factors, non-stomatal factors, or a combination of both [39]. Our study found that stressed ramets significantly reduced g_s_, C_i_, E, while showing an increase in WUE, suggesting that stomatal closure is one of the factors contributing to the decline of P_N_. We speculated that toxic stress damages the root system’s ability to absorb, transport, and store water and nutrients, leading to changes in leaf water content and turgor, and consequently, stomatal closure [40,41]. Observations suggest that clonal integration in connected stressed ramets increases g_s_, C_i_, and E, mitigating the stomatal factors leading to P_N_ reduction.

Our results indicated that the decrease in C_i_ was less pronounced than that in g_s_ and E, alongside a decline in CE, suggesting that CO_2_ availability is not the only limiting factor for photosynthesis. Instead, non-stomatal factors, particularly the reduced photochemical activity of PSII, are likely the primary constraints [11,42]. Chlorophyll fluorescence parameters, such as F_v_/F_m_ and ΦPSII, effectively assess PSII function and the effects of environmental stress. F_v_/F_m_ indicates potential quantum efficiency, while ΦPSII reflects actual efficiency, both revealing damage to PSII [43]. Stressed ramets exhibited significant reductions in F_v_/F_m_ and ΦPSII under heterogeneous stresses, but clonal integration mitigated these declines, highlighting its protective role for PSII reaction centers.

In addition, increases in F_v_/F_m_ are linked to reduced energy loss as heat [44]. Plants utilize protective mechanisms such as NPQ to dissipate excess excitation energy as heat [45]. Our data showed that heterogeneous stresses significantly increased NPQ, whereas clonal integration reduced this increase. A lower NPQ suggests better absorption and utilization of light energy, although it does not directly assess changes in PSII operating efficiency [46,47]. Conversely, F_v′_/F_m′_ estimates the maximum quantum efficiency of PSII photochemistry in illuminated leaves when QA is maximally oxidized [47]. Stressed ramets demonstrated significant reductions in F_v′_/F_m′_, but clonal integration alleviated this impact.

F_0_ indicates fluorescence when PSII reaction centers are open, while q_P_ reflects energy consumed during photosynthesis [44]. Heterogeneous stresses decreased F_0_ and q_P_, but clonal integration significantly alleviated this impairment. An increase in F_0_ and a decrease in q_P_ indicate an over-reduced state of the plastoquinone pool, inhibiting the photosynthetic electron transport chain [48]. ETR, a key indicator of electron transport at the PSII reaction center [47], was negatively affected by heterogeneous stresses; however, clonal integration mitigated this impact. Pollutant toxicity can close or inactivate the PSII system, limiting electron flow for photosynthetic carbon metabolism. Clonal integration alleviated these toxic effects, enhancing the photosynthetic efficiency of connected stressed ramets.

Additionally, F_v_/F_0_, indicating the efficiency of the PSII oxygen-evolving complex (OEC), serves as a sensitive endpoint for assessing photosynthesis in ecotoxicological studies [49]. In stressed ramets, heterogeneous stresses resulted in a decrease in Fv/F0, which is the chlorophyll fluorescence parameter most affected by heterogeneous stress and shows the most significant enhancement due to clonal integration. This indicates that heterogeneous stress can severely damage the structure and activity of the OEC in stressed ramets. However, clonal integration can significantly mitigate this damage.

Metabolic inhibition is another major non-stomatal factor [39]. Oxidative stress negatively affects plant growth. If oxidative stress is not counteracted by the antioxidant system, it can lead to cell membrane damage, protein oxidation, and lipid peroxidation, producing MDA [50]. Antioxidant enzymes mitigate oxidative stress in plants under abiotic conditions, protecting the photosynthetic apparatus and maintaining photosynthetic efficiency [51]. In our study, stressed ramets showed elevated MDA due to heterogeneous stresses; however, clonal integration reduced this increase by enhancing SOD activity and lessening the decline in CAT and POD activities. This suggests that the antioxidant system cannot completely alleviate oxidative damage caused by heterogeneous Pb and/or pyrene stress, but clonal integration mitigates the damage to ramets.

Clonal-integration intensity and the degree of sharing depend on the environmental conditions to which the respective ramets are exposed [18]. A fundamental aspect of a heterogeneous environment is contrast, as minimal differences between patches are essential for promoting resource sharing. When patch contrast is too low, clonal plants may perceive their environment as homogeneous, which diminishes the likelihood of effective clonal integration [52]. Conversely, in heterogeneous environments with higher contrast, the effects of integration are often more pronounced [23,30,52]. However, under severe stress conditions, plants may adopt a strategy of low assistance [18,31].

Our study found that clonal integration can promote growth, but its benefits vary under different heterogeneous stress conditions. Specifically, under heterogeneous Pb+Pyrene stress, the positive effects on connected stressed ramets were minimal. Conversely, the growth, photosynthetic activity, and antioxidant systems of disconnected stressed ramets were most significantly impacted. This suggests that, although the contrast of heterogeneous Pb+Pyrene stress was highest, the promoting effects of clonal integration were notably limited, likely due to the high toxicity of the Pb+Pyrene mixture impacting adaptability. Although heavy metals and PAHs can form cation-π interactions that reduce their bioavailability [4], the ability of Pb and PAHs to form such interactions is relatively weak [53]. As a result, in mixtures of Pb+Pyrene, the reduction in toxicity due to decreased bioavailability is minimal. Additionally, Pb and pyrene may enhance each other’s absorption. Heavy metals and PAHs can damage plant roots by altering the fluidity and lipophilicity of root cell membranes and the ionic balance within cells, making the compromised roots more susceptible to absorbing other pollutants [8,53,54]. Furthermore, heavy metal ions absorbed by plants can be converted into both low-toxicity and high-toxicity forms. However, when PAHs are present, the transformation of heavy metal ions is more likely to favor the production of high-toxicity forms [55]. Additionally, heavy metals can inhibit the mineralization of PAHs within plants [56]. This interaction increases the damage caused by Pb+Pyrene in cells, ultimately affecting the adaptability of clonal integration.

Clonal integration enhanced the growth of stressed ramets, aligning with previous reports. However, it also impacted the growth of donor ramets (unstressed ramets), which contradicts findings from prior meta-analyses suggesting that clonal integration does not affect the growth of donor ramets [57]. Our results indicate that clonal integration influenced not only the biomass accumulation of donor ramets (CPb−, CPy−, and CPP−) but also their photosynthetic efficiency and antioxidant enzyme activity. This finding is consistent with earlier studies on clonal integration under heterogeneous Pb [58] and Cu [59] stress, which noted costs incurred by donor ramets. Notably, in our study, we observed that under the highly toxic Pb+Pyrene treatment, the costs for donor ramets (connected unstressed ramets) were greater than those under individual Pb or pyrene treatments. This, combined with the low benefits associated with clonal integration in the Pb+Pyrene treatment, resulted in the costs of clonal integration for the whole clone exceeding the benefits. Consequently, we identified a phenomenon where clonal plants exhibit high benefits and low costs in low-toxicity environments, while in high-toxicity environments, they display low benefits and high costs. Due to the limitations of our experimental conditions and the stage of this research, we cannot currently determine the underlying reasons for this phenomenon. It may be related to changes in the forms of toxic substances, alterations in absorption and transport capabilities, or impacts on key cellular functions in complex toxic environments, highlighting the need for further investigation into these aspects.

## 5. Conclusions

Our study elucidated the role of clonal integration in modulating photosynthesis, enhancing the efficiency of light energy utilization, and improving antioxidant enzyme activities, thereby supporting biomass accumulation in clonal plants under heterogeneous pollutants. The findings contribute to a deeper understanding of clonal plant behavior in heterogeneous toxic environments. Additionally, our results highlight the variable effects of pollutant toxicity on clonal integration strategies and their impact on donor ramets. Specifically, clonal integration in Zoysia is less effective under combined Pb+Pyrene stress than under individual stressors, with donor ramets experiencing greater effects. Future investigations should aim to comprehensively analyze the interplay between clonal integration and photosynthesis, delving into broader aspects such as the enzymes involved in photosynthetic processes and molecular mechanisms.

## Data Availability

The data that support the findings of this study are available from the corresponding author upon reasonable request.
